# Revalorisation of brewer’s spent grain for biotechnological production of hydrogen with *Escherichia coli*

**DOI:** 10.3389/fbioe.2024.1473704

**Published:** 2024-11-25

**Authors:** Gema Cabrera, José Manuel Jáimez, Jezabel Sánchez-Oneto, Jorge Bolivar, Antonio Valle

**Affiliations:** ^1^ Department of Chemical Engineering and Food Technology, Campus Universitario de Puerto Real, University of Cadiz, Cadiz, Spain; ^2^ Institute of Viticulture and Agri-Food Research (IVAGRO), International Campus of Excellence (ceiA3), University of Cadiz, Cadiz, Spain; ^3^ Department of Biomedicine, Biotechnology and Public Health-Biochemistry and Molecular Biology, Campus Universitario de Puerto Real, University of Cadiz, Cadiz, Spain; ^4^ Institute of Biomolecules (INBIO), University of Cadiz, Cadiz, Spain

**Keywords:** brewer’s spent grain, biohydrogen, *Escherichia coli*, design of experiment, dark fermentation

## Abstract

**Introduction:**

Agro-industrial wastes are generated in huge amounts triggering damages to the environment and human health. Therefore, there is an urgent necessity for its revalorisation into high-value compounds, including biofuels. One such wastes is the brewer's spent grain (BSG), a by-product of the beer industry, which is produced in vast quantities worldwide. The rich-fibre and protein content of BSG makes this waste a valuable resource for biotechnological applications, although the main challenge of this approach is to make the carbohydrates and proteins available for bacterial metabolisation into high-value products. This work aims to optimise a thermal-hydrolysis process to revalorise BSG by bacterial conversion into hydrogen (H_2_), as a clean energy that can replace fossil fuels.

**Methods:**

A 2k full factorial design method was employed hydrolysation of BSG and showed that temperature and acid concentration are significant factors that affect the extraction of reducing sugars (RS) and proteins. Subsequently, steepest ascent and central composite design (CCD) statistical methods were applied to determine the optimal conditions for hydrolysis.

**Results:**

The optimised hydrolysis condition were 0.047 M H_2_SO_4_, 150°C, 30 min and 15% BSG, leading to the theoretical concentrations of 54.8 g RS/L and 20 g/L proteins. However, 5'-hydroxymethylfurfural (HMF) was generated in thermal-hydrolysis conditions at higher temperatures exceeding 132°C. Therefore, a screening of HBSGs fermentation using *Escherichia coli* was conducted in order to identify the most suitable conditions for maximizing H_2_, as well as the production of volatile fatty acids (succinate and acetate) and ethanol. Among the tested conditions, HBSG A17 (117°C, 20 min, and 0.1 M H_2_SO_4_) yielded the highest H_2_ production of 48 mmol/L in this work.

**Conclusion:**

This study provides valuable insights into the optimisation of BSG pre-treatment for biotechnological applications, which may help in the selection of the most appropriate hydrolysis conditions based on the desired end product.

## 1 Introduction

The generation of large quantities of industrial and agricultural waste and the inappropriate disposal of waste have resulted in environmental pollution, emission of greenhouse gases (GHGs) and, consequently, climate change that directly affects human and animal health. For instance, untreated wastes generate unpredictable GHG (CH_4_, N_2_O, etc.) emissions, which can transform into toxic (NOx, SO_2_) or carcinogenic compounds (dioxins, furans, polycyclic aromatic hydrocarbons) (Singh et al., 2021). The application of sustainable, clean and waste-free technologies in industrial processing that add value to waste is imperative for the prudent care of the natural environment and health. To adopt a sustainable approach, bioengineering technologies are becoming increasingly important to improve manufacturing processes based on the fundamentals of a circular economy, increasing their environmental safety and eco-friendliness (Lech and Labus, 2022).

The current massive agricultural and food production leads to large agro-food waste (AFW) generation with enormous handling costs to avoid contributing to environmental pollution. The valorisation of AFW presents a promising opportunity for sustainable energy and bio-based product production through circular biorefineries and the bioeconomy. Advancements in microbial or enzyme engineering and bio-process optimisation are driving significant progress in maximising the efficiency and viability of AFW valorisation into value-added products, such as ethanol, biodiesel, butanol, hydrogen, methane, butyric acid, PHB, etc. (Phitsuwan et al., 2016; Ezeorba et al., 2024). The food industry is a major source of waste with high organic compound content. For instance, lignocellulosic biomass is generated from crops and their subsequent uses in the food industry and is biodegradable; therefore, it represents an advantage over other raw materials due to its availability (Wagner et al., 2021).

Beer is one of the most popular and widely consumed beverages worldwide. According to data from the Barth Haas Group (2023–2024), global production reached 189 million cubic metres. The beer-brewing process starts with the production of the wort, and at the end of this process, the insoluble undegraded part of the barley malt grain or brewer’s spent grain (BSG) is obtained in a mixture with the wort. The wort is filtered through the BSG bed formed at the bottom of the mash, leaving BSG as the main by-product generated from the beer-brewing process (approximately 20 kg of BSG/100 L of beer) (Wagner et al., 2021). BSG is mainly composed of fibre (cellulose, hemicellulose and lignin), proteins, and minerals. Cellulose and hemicellulose are fractions constituted by sugars, among which xylose, arabinose, and glucose are the most abundant; they correspond to approximately half of the dry weight of BSG. Until recently, breweries have usually supplied this material at low cost to local farmers for cattle feed. However, its low price and the high global production of BSG around the world make it an excellent raw material for valorisation (Mussatto, 2014). Due to the presence of carbohydrates and proteins, BSG can be transformed through microbial fermentation into a variety of beneficial products, including enzymes, organic acids, second-generation biofuels, PHAs, prebiotics, sugar alcohols, natural pigments, antimicrobial and bioactive peptides (Mussatto et al., 2006; Lima Moraes dos Santos et al., 2023; Xie et al., 2024). For this, BSG must be hydrolysated to make carbohydrates and proteins available for further application in fermentative processes.

However, the drawback of using BSG is the difficulty of increasing the availability of free sugars to microorganisms because of the resistance to chemical and biological breakdown of lignocellulosic compounds. This effect is caused by several factors, including the crystalline structure of cellulose, the degree of lignification, structural heterogeneity, and complexity of cell wall components, so it must be treated for utilisation of its feedstocks (Guerriero et al., 2016). Several pretreatment techniques have been developed in the last decades to improve the deconstruction of lignocellulosic biomass (LCBs): physical (milling, microwave, etc.), chemical (acid/alkaline hydrolysis, organosolvent processes, etc.), thermochemical (steam explosion, liquid hot water, etc.), and biological (whole cell processes and enzymatic hydrolysis) pretreatments (Baruah et al., 2018).

Chemical hydrolysis pretreatments (acid or alkaline) are the most popular because they lead to a high concentration of fermentable sugars. They do not require special construction materials and are feasible for industrial applications (Singh et al., 2014). However, they generate by-products like furfural, 5'-hydroxymethylfurfural (HMF), phenols, and organic acids (formic or acetic), which can inhibit the subsequent fermentation processes (Wilkinson et al., 2014; Rojas-Chamorro et al., 2020b; Rojas-Chamorro et al., 2020a). In addition, thermochemical pretreatments are highly energy-consuming and typically involve the addition of organic or metallic compounds, which can cause the inactivation of enzymes or the production of adverse toxic effects in the following biological steps. Biological pretreatments require quite a long operating time and are less effective in decomposing recalcitrant biomass (Lech and Labus, 2022). Therefore, it is essential to select the right sequence of pretreatments and the optimal conditions for each to obtain the maximum yield under moderate conditions, that is, avoiding excessive consumption of energy and chemicals (Lech and Labus, 2022).

Pretreatments with dilute acids are the most suitable at the industrial scale as they bring about conversions in an economical and environmental manner. Hydrolysis of BSG with nitric and phosphoric acids has been reported by Rojas-Chamorro et al. (2020b); other studies reveal that diluted sulphuric acid (H_2_SO_4_) is the most extensively used to pre-treat lignocellulosic biomass (Baruah et al., 2018). This approach has been used in several studies as a pretreatment of BSG for the bioconversion of its components for different purposes (bioenergy, biochar, biofuels, and biochemicals). Most studies have been conducted within a range of 1% to 9% H_2_SO_4_ with 5%–30% (w/v) BSG in an autoclave, where the temperature is set at 121°C for 10–30 min. These treatments lead to sugar yields ranging between 0.05 g RS/g BSG and 0.77 g RS/g BSG Plaza et al., 2017; Ravindran et al., 2018; Castilla-Archilla et al., 2021; Corchado-Lopo et al., 2021). This disparity in sugar yields highlights the importance of the conditions used and the type of BSG treated. A treatment with a different temperature was studied by Rojas-Chamorro et al. (2020a). They applied an experimental design to study the factors affecting acid hydrolysis pretreatment with H_2_SO_4_ concentration of 1%–3% (w/v), temperature (110°C and 130°C) and contact time (10–40 min) for hydrolysation of 12.5% BSG (w/v). These results showed that the optimal conditions were 1% H_2_SO_4_, 130°C, and 26 min, obtaining 98% hemicellulose sugar recovery in the liquor and an RS yield of 0.36 g RS/g BSG. Therefore, there is a clear need to determine the most appropriate pretreatment conditions depending on the process to be applied and the product to be obtained, considering different factors related to the circular economy, such as sustainability, economy, and environmental concerns.

The need to replace fossil fuels with more sustainable energy has driven scientists to explore a wide variety of renewable sources. Hydrogen (H_2_) is considered a promising energy carrier because the high energy yield and low heating value of the H-H bond make it a more efficient fuel than hydrocarbon-based fossil fuels (Ferraren-De Cagalitan and Abundo, 2021). H_2_ is produced from a wide variety of sources, although 95% is derived from fossil-based fuels such as oil, natural gas, and coal, releasing GHGs, which are considered drivers of climate change (IRENA, 2018). Compared to conventional methods, biological production of H_2_ (bio-H_2_) offers a more environmentally friendly and less energy-intensive alternative (Ferraren-De Cagalitan and Abundo, 2021). Biological processes for H_2_ production include dark fermentation with bacteria, including *Bacillus sp*., *Enterobacter sp*. *Clostridium* or *Escherichia*, and photofermentation by *Rhodobacter sphaeroides* (Hakobyan et al., 2021). All of them are considered efficient bacteria for H_2_ production (Poladyan et al., 2018). Another approach for bio-H_2_ is microbial electrolysis cells (Lee et al., 2010). Among these methods, dark fermentation is one of the most studied and promising technologies because it is generated at a high rate, and various organic wastes and wastewater can be used as substrates (Ghimire et al., 2015).

In this work, *E. coli* is used for H_2_ production due to its capacity to enhance yields and productivity by metabolic engineering using glucose, glycerol, or different organic compounds contained in waste materials (Valle et al., 2019). In particular, recent publications have demonstrated the feasibility of producing H_2_ using BSG under the following experimental conditions: 4% (w/v) BSG treated with 0.7% (v/v) sulphuric acid in a steam steriliser at 121°C for 20 min, with the pH adjusted to 7.5 and 2.5-fold dilutions of hydrolysed brewer’s spent grain (HBSG) (Poladyan et al., 2018; Mirzoyan et al., 2020). To understand the fermentation of HBSG by *Escherichia coli*, it is essential to know the physiological role of hydrogenases, the enzyme complexes involved in reducing or oxidizing H_2_ when carbohydrates extracted from BSG are metabolised for H_2_ production. In this regard, the studies of fermentation of HBSG by *E. coli* mutants lacking the hydrogenases (Hyd) 1, 2, 3, and 4 demonstrated that Hyd-3 is the principal complex in H_2_ evolution at pH 7.5. On the other hand, Hyd-1 and 2 are responsible for the H_2_ oxidation. Therefore, in order to avoid H_2_ oxidation, the *E. coli hyaBhybC* mutant (which does not express Hyd-1 and 2) was tested for HBSG fermentation at pH 7.5, yielding 40 mL H_2_ (1.7-fold higher than the wild-type strain) (Poladyan et al., 2018). In addition to HBSG, additional strategies combining different carbon sources have also been performed. For instance, HBSG with peptone or glycerol using the mutants described above improved H_2_ when compared to the HBSG medium (Mirzoyan et al., 2020). However, studies about the pre-treatment conditions of BSG for fermentative H_2_ production (FHP) in *E. coli* are insufficient in terms of BSG characterisation reproducibility between BSGs from different brewery industries and pretreatment methods.

The aim of this work is to identify and optimise a chemical pre-treatment process of BSG to obtain hydrolysates (HBSG) rich in assimilable carbohydrates and peptides/proteins for dark fermentation with *E. coli* in a more cost-effective and sustainable process than those described so far. The HBSG was used in various concentrations to formulate a defined HBSG-based medium with the goal of enhancing growth and FHP yields and productivity.

## 2 Material and methods

### 2.1 BSG raw material and chemicals

Brewer’s spent grain (BSG), with an average size of 3–5 cm, was kindly supplied by the “Cerveza Caletera” craft brewery (El Puerto de Santa María, Cádiz, Spain) and dried for 48 h at 50°C for storage and at 105°C for moisture determination. This BSG was obtained from beer fabrication using a mixture of different compositions of malts (Pale Ale MD, Weyermann, and Dingemans). Subsequently, it was ground in smaller particle sizes (Moulinex grinder MC3001, Groupe SEB, France) and stored at room temperature until use. Chemicals such as sulphuric acid (H_2_SO_4_) (95%), hydrochlorhydric acid (HCl) (37%), sodium hydroxide (NaOH), and potassium hydroxide (KOH) were purchased from Panreac Química S.L.U. (Spain). The mineral salts described below were obtained from Scharlab and VWR Chemical; peptone and yeast extract were purchased from Condalab.

### 2.2 Selection of the chemical hydrolysing agent

To determine the capability of the most common hydrolysing agents to make the BSG components accessible, a preliminary experiment was carried out using stock solutions from sulphuric acid (H_2_SO_4_) or hydrochloric acid (HCl) and alkali [sodium hydroxide (NaOH) or potassium hydroxide (KOH)] reagents; water for hydrothermal pre-treatment was used as the reference condition. All the assays were prepared with 50 mL of acid or alkali solution at three concentrations (0.1 M, 0.2 M, and 0.3 M) and 10% (w/v) BSG in screw-capped glass bottles (100 mL) and heated at 121°C for 20 min using an 80 L bench-top autoclave (STERILVAC 80, DAIHAN Scientific, South Korea). After pre-treatment, all experimental assays were cooled at room temperature and the solids were separated from the supernatant by centrifugation at 6,000× g for 10 min. Supernatants labelled as HBSG (hydrolysed brewer’s spent grain) were filtered (by 0.45 µm and 0.22 µm) for subsequent analysis.

### 2.3 Design of experiment for the optimisation of pre-treatment conditions

Once the most appropriate solvent for thermochemical hydrolysis was established, the operating conditions were optimised to increase the availability of sugars and proteins from lignocellulosic material. To this end, a 2k full factorial design was created, including the following factors and levels (in brackets): H_2_SO_4_ concentration (0.05–0.15 M), BSG load (5%–15%) (w/v), contact time (10–30 min), and temperature (102°C–132°C) using an autoclave as previously described to determine which factors significantly influence reducing sugar (RS) extraction. The design resulted in 16 experiments (n = 1) with maximum and minimum levels (+1, −1) and three central points (0) (n = 3) (Table 2). The RS yields were used as response variables, and factors were represented in Pareto chart diagrams.

**FIGURE 1:**
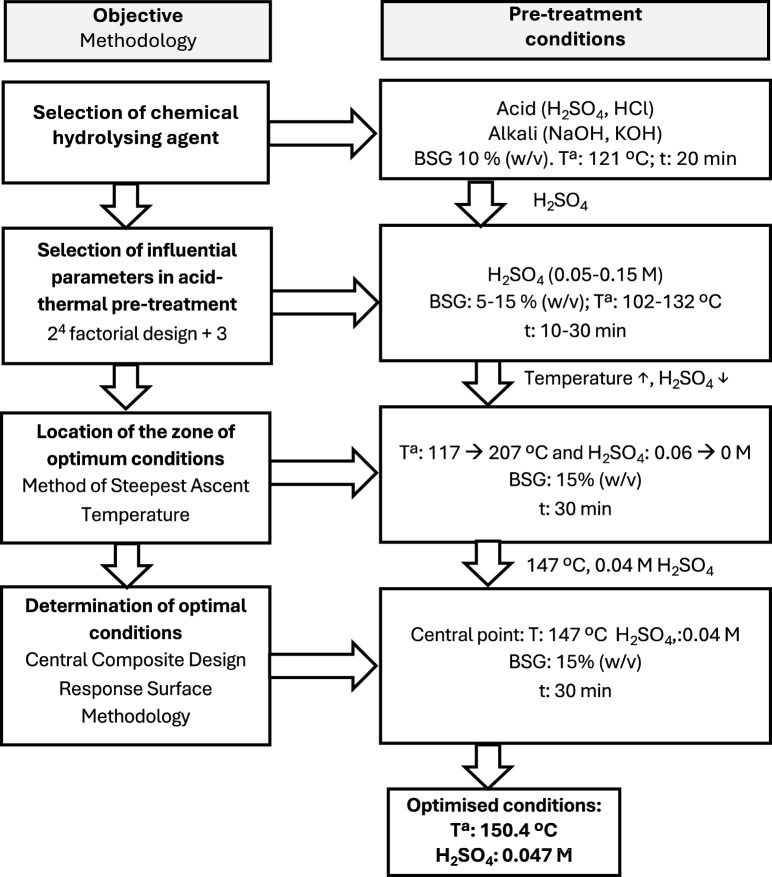
Scheme and description of pretreatment conditions and the design of experiment (DoE) methodology.

Afterwards, to reduce solvent concentration, a full factorial design 2^2^ for two factors (temperature and sulphuric acid concentration) was performed using a 1 L discontinuous stainless-steel reactor (Parr model 4,570 high-pressure reactor) for batch operation to find the optimal region using the method of the steepest ascent of higher temperatures (>132°C) with decreasing acid concentrations (Montgomery, 2017). The reactor has a heating jacket system of 230 V and 400 W covering the vessel. A Pt100 sensor connected to a PID system and placed inside the reactor helps to control and register the temperature. The mixture was heated to the desired temperature, and the corresponding increase in pressure was registered by the ParrCom application. SubW hydrolysis was carried out at temperatures between 117°C–207°C and at acid concentrations from 0.06 to 0 M, 15% (w/v) BSG for 30 min. Subsequently, a two-factor central composite design (CCD) was used to optimise the operational parameters (temperature and acid concentration) of the acid-thermal pre-treatment, and a response surface analysis was conducted to examine the effects of each parameter using RS yields as response variables. The DoE applied in this study used STATGRAPHICS Centurion 19 (v.19.6.03) statistical software. This experimental and statistical procedure is summarised in Figure 1.

### 2.4 Bacterial strain and culture conditions

The *E. coli* BW25113 used was the wild-type strain, purchased from the Keio Collection (Baba et al., 2006). Luria-Bertani (LB) medium was used to reactivate the bacteria from stock 15% glycerol (v/v) and pre-inoculum cultures. The different cultures media were formulated using a minimal medium consisting of phosphate buffer (8.004 g/L K_2_HPO_4_, 3.128 g/L KH_2_PO_4_) and mineral salts: 1.056 g/L NH_4_SO_4_, 0.048 g/L MgSO_4_, 0.009 g/L FeSO_4_, and 5 g/L NaCl. The minimal medium supplemented with 20 g/L peptone (PM) was used for pre-culture in microaerobic conditions. For the hydrogen assays, a minimal medium was formulated with HBSG (HBSG-based medium) at 10%, 15%, 20%, and 40% (v/v) when appropriate. The control assays were performed on an HBSG-based medium with 20% HBSG supplemented with 10 g/L peptone and/or 10 g/L yeast extract. Another control was prepared with minimal medium with 8 g/L glucose and 10 g/L peptone (no HBSG control). All culture media were adjusted to pH 7.5 with NaOH.

The experimental procedure starts by picking up one colony of *E. coli* from an LB-agar plate and growing it in 2 mL LB overnight. A 50 mL PM sample was inoculated with 2% (v/v) overnight pre-culture in 50-mL tubes to raise microaerobic conditions, and bacteria were grown for 3 h. The cells were then centrifuged at 6,000× g for 10 min at 4°C. Inoculation of pelleted cells with optical density (O.D.)∼0.3 was performed in an HBSG-based medium and control media in 12 mL crimp-top vials with 80% headspace sealed with rubber septa. Anaerobic conditions were achieved in a glove box purged with argon to remove oxygen to 6%–8%. Two or more biological replicates were performed and grown for 46 h or 70 h at 37°C in a rotary shaker at 200 rpm.

### 2.5 Analytical methods

For RS quantification, 0.5 mL of the 10-fold diluted samples, adjusted to pH 7, were tested with the dinitrosalicylic acid (DNS) method (Miller, 1959). Protein concentration was analysed following Lowry’s method (Lowry et al., 1951). For qualitative amino acid content, 0.2% ninhydrin stock solution in 95% ethanol was mixed with supernatant samples 1:2 (v/v), heated at 90°C and measured with a spectrophotometer at wavelength 570 nm. For H_2_ quantification, first, the pressure generated (P) was measured in the 9.6 mL headspace vial (V) using the manometer Omega HHP350 and subsequently converted into volume gas (V′) at atmospheric pressure (P′) using the ideal gas law: V′ = (P × V)/P′. Second, relative H_2_ concentration was analysed by gas chromatography (GC) in a gas chromatographer equipped with a Poraplot Q Plot FS 25 × 53 column and a thermal conductivity detector (TCD) (Bruker 450 Daltonik GmbH, Bremen, Germany) following the experimental method (Valle et al., 2015). Carbohydrates in form of monosaccharides (D-glucose, D-xylose, D-arabinose) and disaccharide (maltose), volatile fatty acids (succinate, acetate), and ethanol were quantified in the hydrolysates and in the grown culture’s supernatants and the inhibitor compound (HMF) was quantified in the hydrolysates using high-performance liquid chromatography (HPLC), previously filtered in 0.22 µm. The instrument used was a LaChrom Elite^®^ VWR-Hitachi equipped with an HPX-87H organic acid column (Bio-Rad, Hercules, CA, United States) following the isocratic method of acid water with 5 mM H_2_SO_4_ as mobile phase at 0.6 mL/min during 30 min and a column temperature of 50°C. Ionic chromatography, using the amperometric detector and Metrosep Carb 2 150/4.0 column (Metrohm 930 Compact IC Flex system, Herisau, Switzerland), was performed to discriminate the xylose and galactose peaks that have the same retention time in HPLC and to detect other monosaccharides (D-sorbitol, D-mannitol, D-fructose) or disaccharides (lactose or saccharose). Bacterial growth expressed as cell dry weight per litre (CDW/L) (0.31 g/L = 1 O.D.) was measured with a Merck Spectroquant Pharo 100 spectrophotometer. Crude fibre analysis was performed in HBSG with and without pre-treatment with sulphuric acid and water as a control to determine the contents of lipids, hemicellulose, cellulose, and lignin extracted using the Fibertec™ 8000 (FOSS IBERIA, Barcelona, Spain) and FT 121 Fibertec (FOSS IBERIA, Barcelona, Spain) instruments supported by IVAGRO’s facilities. Prior to the fibre composition analysis, the raw BSG and the solids generated after pretreatments were oven dried at 105°C for 2 h, followed by a 30-min placement in a desiccator at room temperature. The methodology for amylase-treated neutral detergent fibre (aNDF) was based on the Fibertec™ method (ISO 16472:2006), and the determination of acid detergent fibre content and lignin was based on the Fibertec™ method (ISO 13906, 2008). All analyses were performed in triplicate.

### 2.6 Calculation of parameters and statistical analysis

The parameters of the specific production of each compound were calculated as specific H_2_ production (Y_H2/X_) (mmol/g CDW) and volumetric H_2_ production (Y_VH2_) (mmol H_2_/L HBSG-based medium). BSG carbohydrate extraction yields were calculated as g RS/g BSG and g RS/(g BSG·mL of acid). Plots were generated with pro Fit v. 7.0.19 (Quantum Soft, Uetikon am See, Switzerland) software.

## 3 Results

### 3.1 Characterisation and pre-treatment of BSG

**FIGURE 2:**
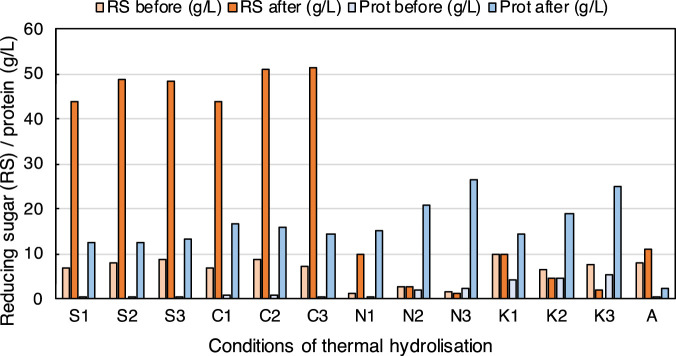
Effect of hydrolysis pre-treatment of BSG. Concentration before and after the pre-treatment in g/L: reducing sugar (RS) and total protein. S1, S2, and S3: 0.1 M, 0.2 M, and 0.3 M H_2_SO_4_, respectively; C1, C2, and C3: 0.1 M, 0.2 M, and 0.3 M HCl; N1, N2, and N3: 0.1 M, 0.2 M, and 0.3 M NaOH; K1, K2, and K3: 0.1 M, 0.2 M, and 0.3 M KOH and A: water. The data from each condition are obtained from one replicate and are shown in Supplementary Table S3.

BSG was dried at 50°C for 48 h until it lost 95% of its moisture content. It was subsequently ground, and only the fraction smaller than 1.7 mm was selected by sieving (Supplementary Tables S1, S2). Acid-, alkali-, and hydrothermal processes are reported in the literature. This work tested four chemicals for the pre-treatment of BSG to select the most efficient and least expensive method for carbohydrates and protein extraction. For this, the hydrolysis of BSG with acids (H_2_SO_4_ or HCl) or alkalis (NaOH or KOH) at three concentrations was applied, followed by a thermal stage (121°C, 20 min). After centrifugation to separate the solid fraction, the liquid fraction (hydrolysate or HBSG) of each assay was neutralised. The reducing sugars (g RS/L) and proteins (g/L) were analysed in the liquor (Figure 2; Supplementary Table S3). The RS increased from 43.8 g/L to 48.8 g/L with increasing sulphuric acid concentrations (0.1–0.3 M) and from 44 g/L to 51.4 g/L with increasing hydrochloric acid concentration. These pretreatments showed a significant RS extraction with respect to those conditions after acid addition at time zero, that is, before thermal treatment (6.8–8.7 g RS/L) and with respect to water control before (7.94 g RS/L) and after (11.18 g RS/L) thermal treatment. In the case of pre-treatment with alkalis, the behaviour is different. When KOH was used, the concentrations of RS at time zero were very similar to those obtained with acids and water; however, after applying the thermal stage, no increased RS content was observed for the lowest concentration (K1: 0.1 M), and for higher base concentrations (K2 and K3), RS decreased. The tests with NaOH have the particularity that at the initial time, the sugar concentration is very low, and only in the case of N1 (0.1 M NaOH) does it reach a concentration similar to that obtained in the pre-treatment with water. Therefore, with regard to the concentration of sugars in the hydrolysate, acid-thermal pre-treatment is much more effective than alkali or water pre-treatment (Figure 2). Regarding protein extraction, acid pretreatments yielded a total protein of approximately 13 g/L with sulphuric acid and between 14.3 g/L and 16.7 g/L with hydrochloric acid. In the case of alkali, protein extractions were higher: 15.2–26.5 g/L with NaOH and 14.6–25.05 g/L with KOH. In both conditions, a positive correlation between extracted proteins and alkali concentrations was observed. As previously mentioned, the reference assay with water also showed very low protein extraction (Figure 2).

On the other hand, in the case of the hydrolysated BSG (HBSG) with the highest RS content, the use of acids was more appropriate than alkali for further fermentative processes because the pretreatments with any concentration of acids showed similar protein concentration to that of 0.1 M alkali treatment. To select the most appropriate acid concentration of those studied, the RS yield parameters were calculated with respect to the volume and costs of acids (Table 1). The RS yields were significantly higher when 0.1 M acid was used (approximately 7.9 g RS/mL acid in both cases) than those obtained with 0.2 M or 0.3 M. Nonetheless, the costs of HCl with respect to kg of RS are approximately 50% higher than those obtained with H_2_SO_4_. The higher RS yield is very interesting when either of these two acids was used. However, it is more reasonable to use this acid (Table 1) because the 5 M HCl stock solution requires a larger volume of commercial acid due to its molecular weight, and the cost of such commercial HCl is higher than H_2_SO_4_ (Supplementary Table S4). To avoid the excessive use of chemicals, the pre-treatment with H_2_SO_4_ at the lower concentration was selected to continue optimising the pre-treatment of BSG and develop a more economical and sustainable process.

**TABLE 1:** RS, reducing sugar; S, sulphuric acid (H_2_SO_4_); and C, hydrochlorhydric acid (HCl). The calculation of acid costs is described in Supplementary Table S4. Data are obtained from one replicate.

	Acid	RS (g/L)	g RS/mL acid	Acid cost (€/kg RS)
S1	0.1 M H_2_SO_4_	43.73	7.88	1.47
S2	0.2 M H_2_SO_4_	48.75	4.39	2.64
S3	0.3 M H_2_SO_4_	48.27	2.90	4.00
C1	0.1 M HCl	44.08	7.95	2.20
C2	0.2 M HCl	51.03	4.60	3.80
C3	0.3 M HCl	51.38	3.09	5.65

### 3.2 Design of experiment for pre-treatment of BSG and characterisation of HBSG

**FIGURE 3:**
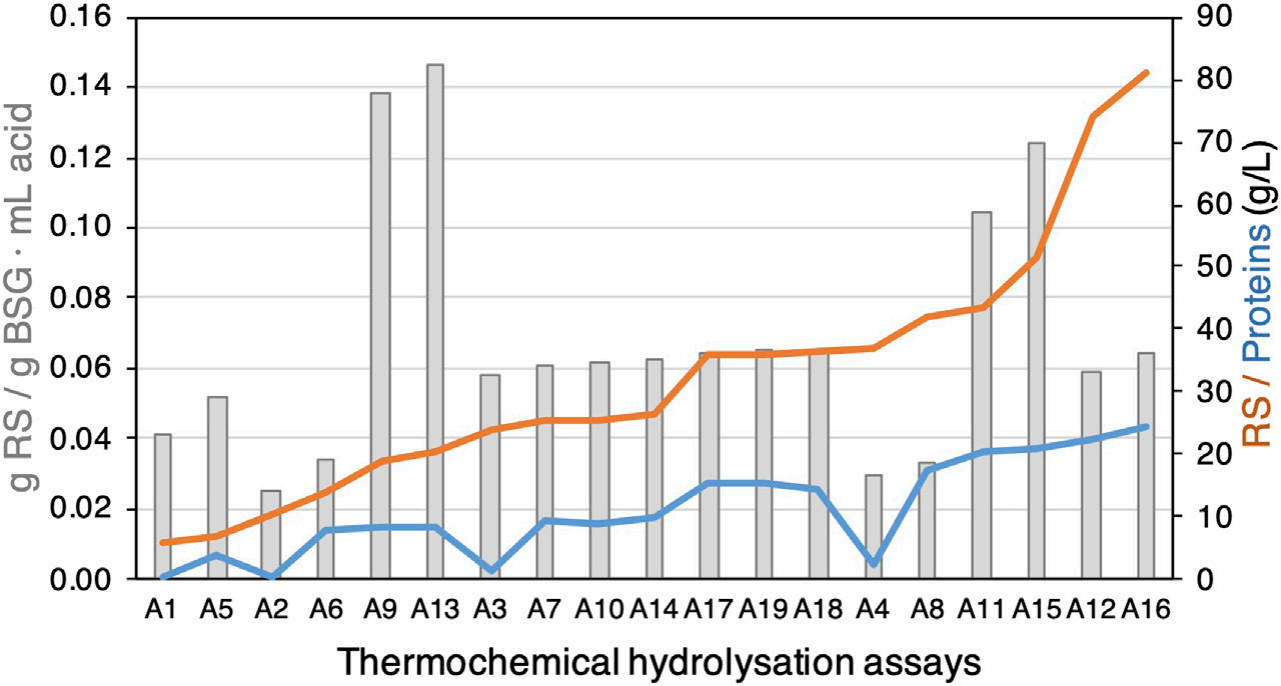
Curves of reducing sugar (RS) and proteins (g/L); box plot of RS yield with respect to BSG and acid used. All results were obtained under 17 thermochemical conditions using one replicate, as described in Table 2 and Supplementary Table S5. A17, A18, and A19 are plotted independently but are considered as three replicates.

Based on the previous results, a 2^4^ factorial experimental design was carried out to determine the most influential factors in the acid hydrolysis of BSG, which were: (A) H_2_SO_4_ concentration (M), (B) BSG load (%w/v), (C) contact time (min), and (D) temperature (°C). The experimental design consisted of 19 experiments, including three centre points. The experimental matrix with the values of each parameter is presented in Table 2, and the experimental results in terms of g RS/L, g protein/L, and yield parameters are represented in Figure 3 (data reported in Supplementary Table S5). The core temperature was selected considering that 132°C is the maximum temperature that can be reached in a laboratory autoclave (A). At first glance, a positive correlation can be observed between temperature and RS concentration and yield together with protein extraction. For instance, the A16 condition showed the highest RS extraction (80.9 g/L RS and 0.54 g/g BSG) and also the highest protein extraction (24.45 g/L). However, if the analysis considers RS yield with respect to acid as a response variable, the experiments with more diluted acid (0.05 M) presented the best results (A9 and A13 with 0.14–0.15 g RS/g BSG mL), although the concentrations of RS and proteins are lower. Therefore, the most interesting conditions are A12 and A16 in terms of RS concentrations, but if we also consider the RS yield, the A11 and A15 are more notable. In addition, the protein extraction in these conditions (A11, A12, A15, and A16) is higher than 20 g/L and, with respect to the other conditions (Figure 3), showed similar results when using 0.2 M NaOH or KOH (Figure 2). In contrast, the conditions with the lowest RS concentration and yields are A1, A2, A5, and A6 when the temperature (102°C) and % BSG (5%) are lower, so temperature seems to be an important factor that affects these parameters.

**TABLE 2:** Full factorial design (2^4^) analysis using four factors: A, concentration of sulphuric acid; B, percentage of BSG in w/v; C, contact time of acid with BSG; and D, temperature. Three central points are A17, A18, and A19.

Exp	A	B	C	D	A [H_2_SO_4_] (M)	B BSG (%w/v)	C Time (min)	D Temp. (°C)
A1	−1	−1	−1	−1	0.05	5	10	102
A2	1	−1	−1	−1	0.15	5	10	102
A3	−1	1	−1	−1	0.05	15	10	102
A4	1	1	−1	−1	0.15	15	10	102
A5	−1	−1	1	−1	0.05	5	30	102
A6	1	−1	1	−1	0.15	5	30	102
A7	−1	1	1	−1	0.05	15	30	102
A8	1	1	1	−1	0.15	15	30	102
A9	−1	−1	−1	1	0.05	5	10	132
A10	1	−1	−1	1	0.15	5	10	132
A11	−1	1	−1	1	0.05	15	10	132
A12	1	1	−1	1	0.15	15	10	132
A13	−1	−1	1	1	0.05	5	30	132
A14	1	−1	1	1	0.15	5	30	132
A15	−1	1	1	1	0.05	15	30	132
A16	1	1	1	1	0.15	15	30	132
A17	0	0	0	0	0.1	10	20	117
A18	0	0	0	0	0.1	10	20	117
A19	0	0	0	0	0.1	10	20	117

**FIGURE 4:**
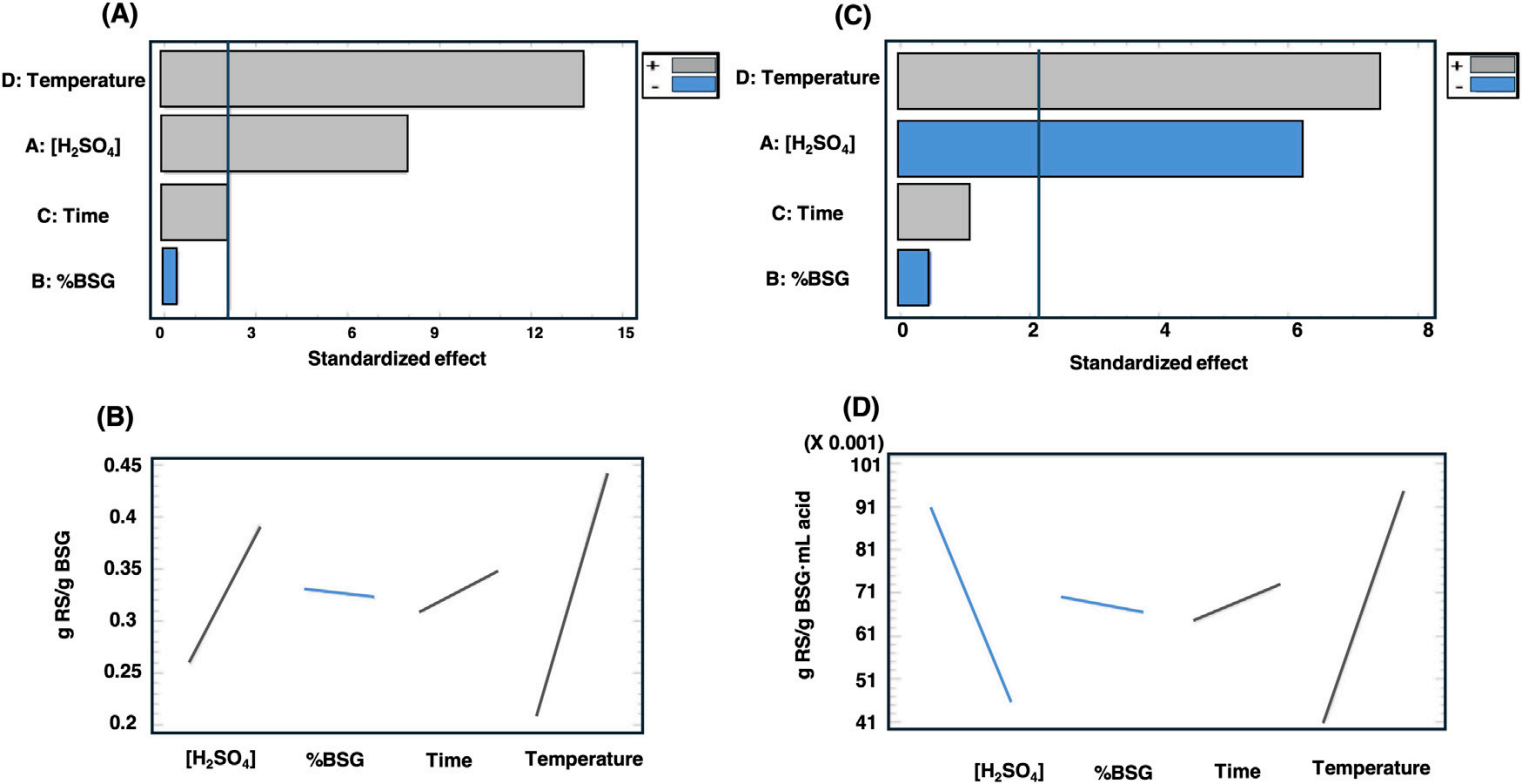
Standardised Pareto chart of RS yields as response variables: g RS/g BSG **(A,B)** and g RS/(g BSG mL acid) **(C,D)**.

To this end, Pareto chart statistical analysis was carried out using both RS parameter yields (g RS/g BSG and g RS/g BSG mL acid) as response variables (Figure 4). The results indicate that temperature has a positively statistically significant influence in both response variables; in contrast, the percentage of BSG treated and contact time have no statistically significant influence on RS extraction. However, the content of H_2_SO_4_ has a significant positive influence when the g RS/g BSG is analysed (Figures 4A, B, respectively) but a negative influence when the g RS/g BSG mL acid is analysed (Figures 4C, D, respectively). This is an important parameter to consider when optimising the process for sustainability, as RS yields depend on temperature and sulphuric acid.

To further disclose the real effect of thermochemical hydrolysation through BSG lignocellulosic material, a fibre analysis was performed in BSG with and without pretreatment, as described in Section 2.5 (Table Supplementary Table S6). The hydrolysation A15 and A16 experiments could not extract lipids and cellulose because the relative concentrations were higher with respect to the pre-treatment with water. However, the hemicellulose decreased by approximately 30% with respect to the control in water, indicating that temperature and acid provoke hydrolysation of hemicellulose, increasing carbohydrates available in the HBSG (Figure 3; Supplementary Table S5).

### 3.3 Optimisation of thermochemical pre-treatment of BSG

**FIGURE 5:**
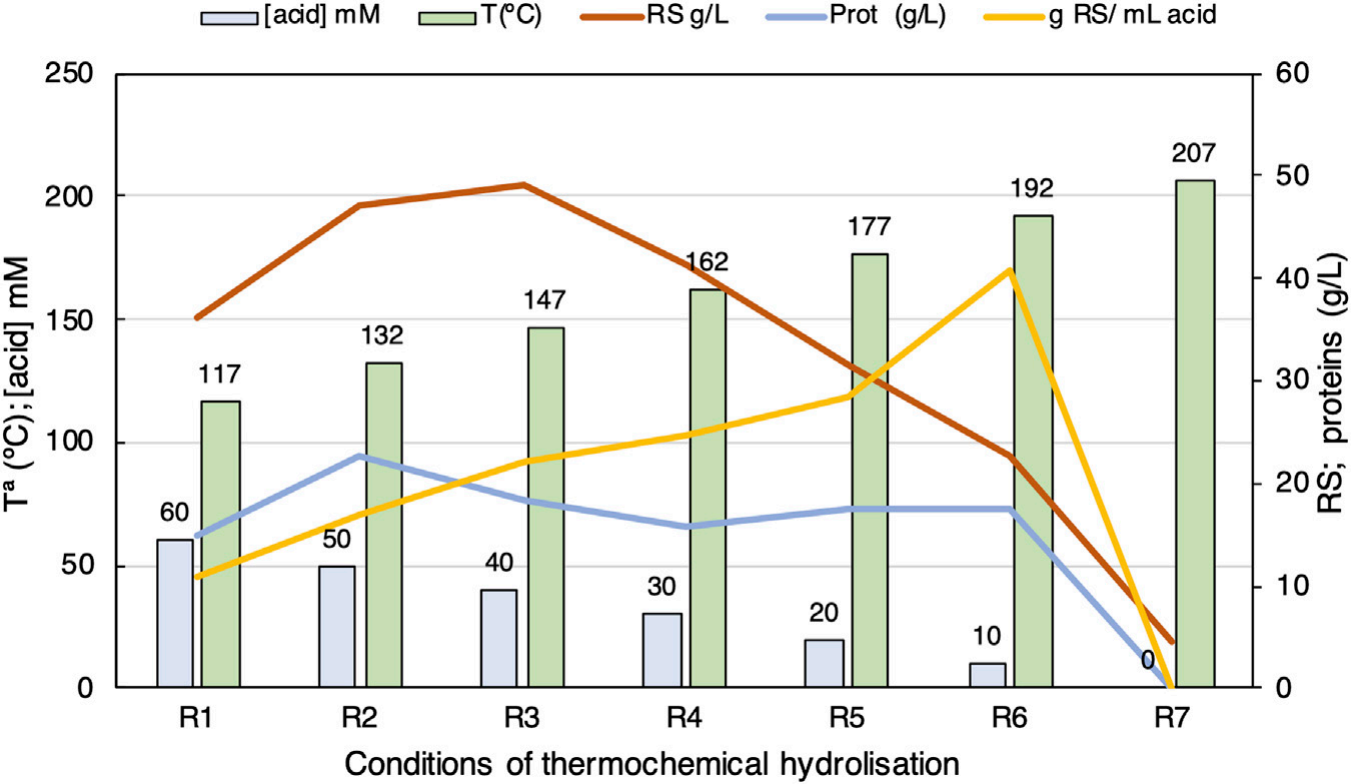
Curves of RS, proteins, and total carbohydrates analysed by HPLC (g/L). Box plots of temperature and sulphuric acid concentration obtained from HBSG samples in seven different thermochemical conditions using one replicate, as indicated in Supplementary Table S8.

The results obtained previously indicate that temperature and H_2_SO_4_ concentration can be studied as factors using the RS yield parameter (g RS/mL acid) as a response variable. In all of the experiments, BSG was fixed at 15% (w/v), and contact time was fixed at 30 min because RS yields were higher (Supplementary Table S5). Then, a set of experiments was designed consisting of a temperature increase to 207°C concomitant with a steep increase of sulphuric acid concentrations (Figure 5; Supplementary Table S7). Because the autoclave cannot arise temperatures higher than 132°C, a high-pressure stainless-steel reactor was used. To test the applicability of both equipment setups for hydrolysis, the assay in an autoclave (A) was carried out under the same conditions (R2 = A15) in the stainless-steel reactor. The results obtained for RS extraction showed that the optimum could be around the R3 condition (147°C, 0.04 M), but in the case of protein extraction, the R2 condition is slightly better. However, the RS yield (g RS/mL acid) increased linearly with temperature. Meanwhile, the acid concentration diminished, so the R6 condition showed the highest RS yield (40.93 g RS/mL acid), although the RS was significantly lower (22.71 g/L). Although it is interesting to use better yield conditions, it is noteworthy that higher temperatures involve higher energy consumption and additional time to reach these thermal conditions. In addition, the RS concentration diminished considerably, possibly due to the thermal degradation of carbohydrates into HMF, which is a toxic compound.

**FIGURE 6:**
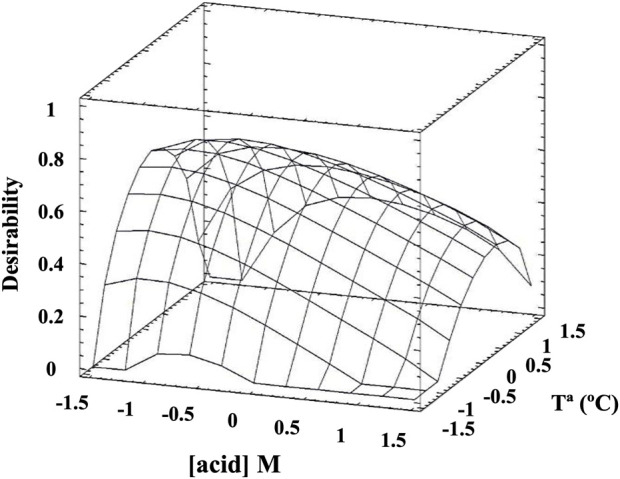
Response surface curve for the optimisation of RS concentrations as a function of sulphuric acid and temperature.

A central composite design of experiments (CCD) was used to study the effect of two factors, H_2_SO_4_ concentration and temperature, on the RS (g RS/L) and protein (g protein/L) concentrations as response variables, using a multiple response optimisation methodology to determine the combination of experimental factors that would maximise both responses simultaneously. For this, R3 has been considered the central point to establish the optimal pre-treatment conditions. The R2 and R4 conditions were also included in the CCD. Therefore, a matrix for two factors of CCD analysis generated 11 assays (OP1–OP11) (Supplementary Table S8). All hydrolysis assays were performed as previously described with 15% (w/v) BSG and a contact time of 30 min. The experimental data obtained were used to determine the response surface curve (RSC) that represented acid concentration, temperature, and the desirability function to estimate this effect. Analysis of the experimental data revealed that run OP5 of the experimental design achieved the maximum desirability (0.661302). Further analysis identified the optimal combination of factors to maximise the desirability functions: [H_2_SO_4_] = −0.695947x + 0.040 (x = −0.01) and for temperature (°C) = −0.22771y +147 (y = 15), which is 0.047 M acid and 150.4°C, respectively. This factor combination is expected to produce 54.80 g RS/L and 20.72 g protein/L (Figure 6). These results confirm that the optimal point is very close to the central point (R3) (0.04 M H_2_SO_4_ at 147°C) (Figure 5). The concentration of RS in the optimised conditions is significantly lower than previously obtained in A16 (80 g RS/L); however, a 4-fold higher concentration of H_2_SO_4_ (0.15 M) was needed than in the optimised condition (0.04 M). The advantage of this optimisation is the lower consumption of acid, but the disadvantage is that it requires a higher temperature than the conventional autoclave can provide, so it would need a high-pressure reactor.

To select the best conditions for the hydrolysation of BSG for fermentative processes with bacteria, 5'-hydroxymethylfurfural (HMF) was analysed in all of the hydrolysates: (A1–A17), (R1–R7), and (OP1–OP11) (Supplementary Tables S5, S7, S8, respectively), because this compound is produced by the degradation of sugars at high temperatures. The results indicate that HMF (mg/L) is produced when an autoclave was used only in some conditions at 132°C (A10, A11, A13–16). Conditions A15 and A16 obtained the highest concentrations, ∼11 and ∼16 mg/L, respectively. However, when higher temperatures of 147°C were used, higher HMF concentrations were detected; for instance, 211 mg/L HMF and 282 mg/L HMF at 177°C and 192°C, respectively. In contrast, a lower concentration (170 mg/L) was generated at 207°C without sulphuric acid (Supplementary Table S8). In addition to high temperature, sulphuric acid has a significant influence on HMF formation.

### 3.4 Study of the effect of HBSG on hydrogen production

Based on the highest RS titre and yield of HBSG, the A16 condition was selected to formulate the HBSG-based medium with mineral salts and the addition, in some cases, of peptone and/or yeast extract as reference media to evaluate FHP in *E. coli* wild-type strain. The volumetric hydrogen production (Y_VH2_) at 46 h was evaluated and revealed how the increase of HBSG concentration, 10%, 15% and 20% (v/v), that theoretically contained 8 g/L, 12 g/L, and 16 g/L of RS, respectively, increased H_2_, with values of 10 mmol/L, 25 mmol/L, and 32 mmol/L, respectively (Table 3). When peptone and/or yeast extract were added to the HBSG-based medium, H_2_ titres were lower than those obtained with 20% HBSG. In fact, the values obtained with only peptone are not significantly different than those obtained with peptone and yeast extract. However, when only yeast extract was used, lower values were obtained. It seems that the additional N-sources are not necessary to increase H_2_ synthesis. On the other hand, 8 g/L glucose with peptone was also used as reference media and obtained similar values to those obtained with 20% HBSG. This preliminary indicates that HBSG contains similar amounts of nitrogen and carbon as those in a defined medium with glucose and peptone.

**TABLE 3:** Volumetric hydrogen production (Y_VH2_), expressed in average and standard deviation using n = 3–6 replicates in *Escherichia coli* wild-type strain using 10%, 15%, and 20% HBSG (v/v) and glucose (8 g/L) as carbon source and additionally peptone (P) (10 g/L) and/or yeast extract (Y) (10 g/L) at 46 h.

Condition	Y_VH2_ (mmol/L)
10% HBSG	12.40 ± 0.30
15% HBSG	26.80 ± 2.01
20% HBSG	33.96 ± 2.67
20% HBSG + P	32.46 ± 2.27
20% HBSG + E	22.50 ± 1.28
20% HBSG + PE	29.90 ± 2.51
Glucose (8 g/L) + P	41.10 ± 2.03

**FIGURE 7:**
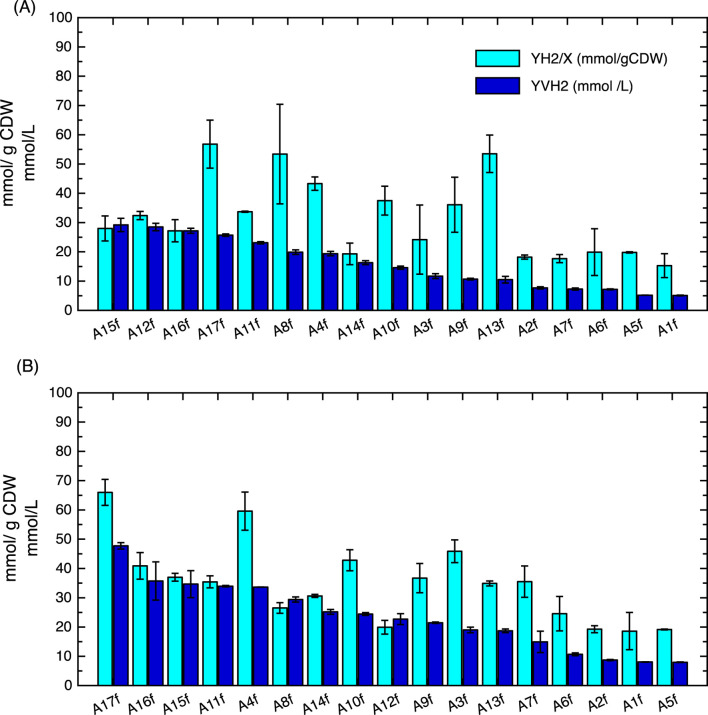
Specific H_2_ production (Y_H2/X_) and volumetric H_2_ production (Y_VH2/X_) in *Escherichia coli* wild-type strain using 20% **(A)** and 40% (v/v) **(B)** from HBSG samples obtained under 17 different conditions, as described in Table 2, at 70 h after inoculation. Plots represent the average, and error bars represent the standard deviation using two biological replicates.

According to this, it was proposed to increase the HBSG concentration for FHP in *E. coli* to evaluate the viability of the HBSG pretreatments from 2k design in an autoclave (A1–A17) (Supplementary Table S5) at 20% and 40% (v/v) (Figure 7). When 20% HBSG was used, conditions A12f, A15f, A16f, and A17f produced 25.7–28.2 mmol/L, and no significant differences were found between them. However, the Y_H2/X_ using A8f and A13f was approximately 53.4 mmol H2/g CDW and 56.8 mmol H_2_/g CDW in A17f, indicating that the growth was lower and the production was more efficient than the other conditions (Figure 7A). In the case of hydrolysated assays at 40%, A17f produced the highest volumetric and specific H_2_ production with 47.7 ± 1.1 mmol/L and 62.8 ± 4.4 mmol/g CDW, respectively, which are significantly higher than the rest of the conditions. However, A4f, A11f, A15f, and A16f produced between 33.6 mmol/L and 35.7 mmol/L, which is higher than those obtained using 20% HBSG (Figure 7B). In A11f, A15f, and A16f, more than 40 g/L of RS in the hydrolysates and 20 g/L of proteins were obtained at 132°C, but in the case of A4f, approximately 37 g of RS/L and 2.34 g/L of proteins were obtained. In contrast, in the cases of A1f, A2f, and A5f–7f, the yields with 20% and 40% were lower than 15 mmol/L, which are presumably associated with lower carbohydrate concentrations than 7 g/L, which could limit the biomass growth (<0.5 g CDW/L). In contrast, the H_2_ values of A15f, A16f, and A17f were significantly higher, at 38 mmol/L, 40.3 mmol/L, and 48.5 mmol/L in 40% HBSG, respectively, which is likely due to higher sugar concentration; biomass growth yields were 1.02 CDW/L, 0.98 CDW/L, and 0.73 g CDW/L, respectively (Supplementary Figure S1). The control experiment in a minimal medium with glucose and peptone showed 41 mmol H_2_/L, indicating that, apart from carbohydrate concentration, other factors can affect growth and H_2_ metabolism, such as protein hydrolysation in peptides or free amino acids, inhibitory compounds, cofactors, etc.

**FIGURE 8:**
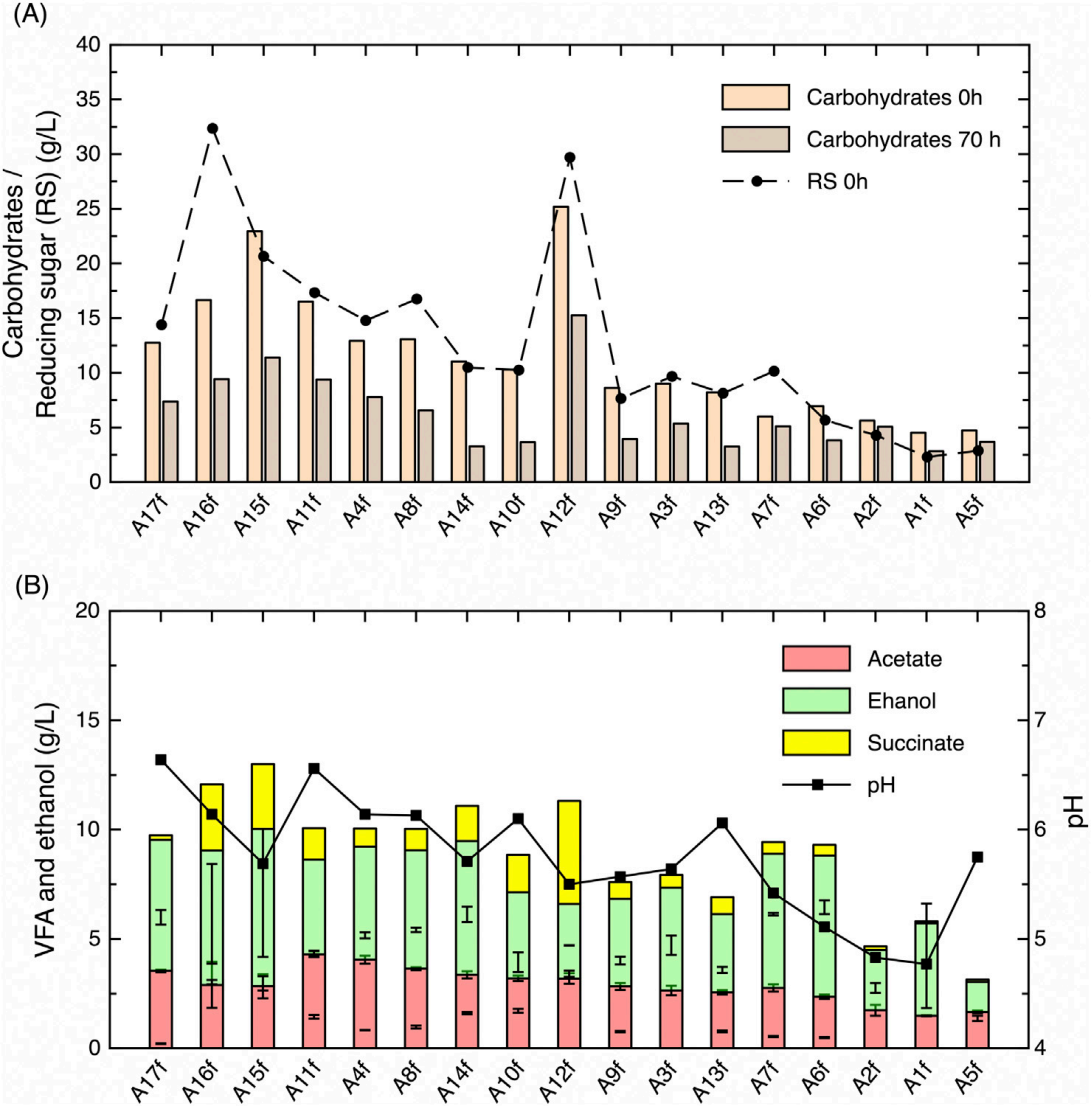
Analysis of the fermentative HBSG samples (A1–A17) in *Escherichia coli* using 40% (v/v) at initial (0 h) and at 70 h post-inoculum of carbohydrates and RS (g/L) **(A)** and VFA (acetate and succinate), ethanol, and pH at 70 h **(B)**. Plots represent the average, and error bars represent the standard deviation using two biological replicates.

The carbohydrates maltose, D-glucose, D-xylose, and D-arabinose were analysed by HPLC and found to be produced in these hydrolysation process conditions (Supplementary Table S9). On the other hand, D-sorbitol, D-mannitol, D-galactose, D-fructose, lactose, cellobiose, and saccharose were negligible as detected by ionic chromatography (<0.1 g/L) (data not shown). The fermentative volatile fatty acid (VFA) compounds (acetate and succinate) and ethanol produced by the bacteria in the HBSG-based media at the initial time and 70 h after inoculation were analysed (Supplementary Table S10). First, carbohydrate concentrations correlate with RS in all of the HBSG conditions except A16; more RS than carbohydrates was detected. This could be explained because “other carbohydrate” was not identified by HPLC. Surprisingly, significantly more RS was detected in HBSG A12 at initial conditions with approximately 25 g/L and 30 g/L, respectively (Figure 8A). In the A17f, A16f, and A15f conditions, carbohydrates at the initial period were approximately 13, 17, and 23 g/L, respectively, and also correlated to VFA and ethanol production amounts generated after hydrolysation and fermentative production that were approximately 10, 12 and 13 g/L, respectively (Figure 8B). These data show an inverse correlation between volumetric H_2_ production and VFA + ethanol; for instance, A17f produces 47.7 mmol H_2_/L and 10 g/L VFA, which is different from that obtained in A16f, which was 35.8 mmol/L and 12 g/L VFA. It is evident that fermentative metabolic pathways compete between them to balance biomass growth. The production of H_2_ and VFA in the conditions A11f, A15f, and A16f are associated with *E. coli* growth that was higher than 0.9 g CDW/L; however, the fermentation with A8f and A12f allowed the growth of more than 1 g CDW/L, but H_2_ production with A8f was lower than 25 mmol/L. Surprisingly, in fermentation with A17f, *E. coli* produced more H_2_, but it was not associated with growth. The amount of *E. coli* was less than 0.7 g CDW/L, so in this case, the growth biomass compromises the H_2_ synthesis and VFA. The pH levels in all of the fermented HBSG samples were higher than 5.5, except for the ones that produced lower H_2_ and VFA (HBSG A1f, A2f, A6f, and A7f) (Figure 8B). The temperature used in these conditions was 102°C, so the extractions of sugars and proteins were significantly lower than the other conditions that used higher temperatures. In contrast, the A15f and A16f conditions were pre-treated at 132°C and 30 min, and even though they were more efficient in extracting carbohydrates for FHP, fermentation with A17f (pre-treated at 117°C for 20 min) produced the highest volumetric H_2_ production (48 mmol/L).

The carbohydrates were not completely consumed in any of the HBSG samples used. Approximately 11.6 g/L remained in the case of A15f after a fermentation that consumed 50%. In the case of A16f and A17f, the consumption was even lower, with approximately 42% of carbohydrates consumed. On the other hand, protein and amino acid concentrations after fermentation decreased in A15f but not in either A16f or A17f, indicating that the consumption by *E. coli* is only performed in A15 (Supplementary Figure S2). However, in this condition, protein and amino acid consumption does not significantly affect growth or FHP, so it would be necessary to further analyse the nitrogen source or the assimilable amino acids/proteins in the HBSG-based media.

Ethanol is produced from fermentation but is also produced by hydrolysation, obtaining up to 7.2 g/L in A15f and 6.2 g/L in A16f and A17f. Acetate was produced in A15f and A16f (∼2.9 g/L) and in A17f (3.5 g/L). Succinate was slightly produced in A15f and A16f (∼3 g/L) but was negligible in A17f. It is noteworthy that A12f, which contains a high concentration of carbohydrates concomitantly with high growth, produced less H_2_ titre (22 mmol/L) than A15f–A17f, so metabolisation of the carbon source favours succinate (4.7 g/L) instead of H_2_ synthesis (Figure 8B; Supplementary Table S10).

## 4 Discussion

The key point in the effective use of the valuable components of BSG is the appropriate selection of processes that enable their successful extraction from this raw material. The main BSG pre-treatment conditions reported so far are mechanical following thermochemical hydrolysis and, in some cases, combined with biochemical processes. Although chemical treatment has the most negative impact on the environment, it is still the most commonly used (Lech and Labus, 2022). This work showed that extraction of carbohydrates is more efficient when acids are used instead of alkalis, mainly due to higher efficiency, as previously reported (Wilkinson et al., 2014). These facts are the main reasons for the intensification of the search for more ecological methods of pre-treatment. In this case, focussing on the development of efficient mechanical and thermal pre-treatment processes seems to be a promising solution to problems like by-product formation and aggressive chemical utilisation; for instance, ball milling, autohydrolysis, ultrasound pre-treatment, steam explosion, subcritical water hydrolysis, or even nonthermal plasma treatment. These methods result in a lower concentration of sugars but do not lead to undesirable by-products. Therefore, minimizing the use of chemicals for BSG pre-treatment methods, with a strong focus on upgrading their efficiency in the conversion of cellulose and hemicellulose (Lech and Labus, 2022), has been established in this work.

For this, the H_2_SO_4_ solvent was first selected due to the higher BSG yield obtained and lower cost (Table 1), but a 2k design was conducted to identify the most significant parameters for BSG hydrolysation. The factors that have a significant influence on RS yields as response variables were temperature and acid concentration. Hydrolysation of BSG with H_2_SO_4_ has been reported by several authors (Plaza et al., 2017; Rojas-Chamorro et al., 2020a; 2020b; Castilla-Archilla et al., 2021) who have used a range of 0.7%–9% acid at 121°C or 130°C for 15–30 min; these are similar to the pre-treatment conditions used in this work. Poladyan et al. (2018) used 0.7% (v/v) at 121°C for 1 h of pre-treatment. Rojas-Chamorro et al. (2020a) reported that the content of carbohydrates was 44.5 g/L when using the conditions of 1% v/v H_2_SO_4_, 130°C, 26 min and 12.5% (w/v) BSG, which was lower than the results obtained in A16 (0.15 M = 0.8% v/v, 132°C, 30 min and 15% (w/v) BSG, which produced 80 g RS/L (Figure 3; Supplementary Table S5). A similar result was obtained by Plaza et al. (2017), who obtained (47 g/L), using 1% (v/v) H_2_SO_4_, 121°C and 20 min with 15% (w/v).

These similar results can be explained by determining cellulose and hemicellulose compositions, which were 7% and 20%, respectively, very similar to those obtained by Santos et al. (2003), who reported 9% and 19%, respectively. Other authors have reported similar values of cellulose (9%–25%) and hemicellulose (19%–43%) (Emmanuel et al., 2022; Lech and Labus, 2022). Cellulose can be converted into glucose by chemical hydrolysis, while hemicellulose can be converted into xylose, glucose, and arabinose (Mussatto, 2014). These results show that carbohydrates can be extracted from hemicellulose content; however, the relative concentrations (%) have a wide deviation. The composition of BSG is very variable and depends on the type of grain, method of preparation, and mashing procedure (Santos et al., 2003; Mussatto et al., 2006), although the pre-treatment methods used in this study allow comparison with published studies.

The RS yields (g/g BSG) obtained in several HBSG conditions were 0.34 and 0.36 in A15 and A17, respectively, which are similar to the 0.36 g/g BSG reported by Rojas-Chamorro et al. (2020b), but A16 yielded a higher amount of 0.54 g/g BSG. Furthermore, the amounts of H_2_SO_4_ used were 0.3% (=0.05 M), 0.8 (=0.15 M), and 0.6% (0.1 M) in A15, A16, and A17, respectively (Table 4); therefore, the RS yields with respect to the acid used (g RS/g BSG mL acid) were also higher than those reported by Rojas-Chamorro et al. (2020b) and Plaza et al. (2017).

**TABLE 4:** Summarised HBSG pretreatment conditions with high reducing sugar (RS) yield (g RS/g BSG) in the standardised conditions of 15% (w/v) BSG and contact times of 20 min or 30 min using one replicate are shown on the left. HMF, 5'-hydroxymethylfurfural. On the right are shown H_2_ titre, VFA (succinate and acetate), and ethanol concentration values obtained at central levels. Carbohydrate consumption and percentages of HBSG used in the fermentation process are expressed as averages. Additional reference value data are shown from the literature. ND, not determined.

BSG pre-treatment						Fermentation with HBSG medium				
Assay	Acid % (v/v)	T^a^ (°C)	RS (g/L)	RS yield (g/g BSG)	HMF (mg/L)	HBSG (%) (v/v)	Carb consumption (g/L)	Y_VH2_ (mmol/L)	VFA (g/L)	Ethanol (g/L)
A15	0.3	132	51.63	0.34	10.9	40	11.5	37.9	3.0 2.8	7.2
A16	0.8	132	80.88	0.54	15.9	40	7.3	40.3	3.0 2.9	6.2
A17	0.6	117	36.18	0.36	<0.001	40	5.3	48.5	0.2 3.5	6.0
Poladyan et al. (2018)	0.7	121	ND	ND	ND	4	ND	1.5	ND	ND
Rojas-Chamorro et al. (2020a)	1	130	44.5	0.36	ND	5	43.4	ND	ND	17

RS and proteins have been extracted from lignocellulosic material across a range of temperatures (117°C–147°C), with high yields in the OP5 and the optimised conditions, with respect to BSG and acid consumption (Supplementary Table S8). These yields are also higher than those reported by Plaza et al. (2017) and Rojas-Chamorro et al. (2020b). A limited range of temperature (132°C–150°C) and reduction of H_2_SO_4_ consumption <1% (v/v) (1% = 0.18 M) is a promising strategy for saving reagent costs without compromising RS yield. Nevertheless, HMF is produced at temperatures higher than 121°C. In high concentrations, HMF is toxic for bacteria and impair their viability and consequently decreases the efficiency of fermentative processes (Lech and Labus, 2022). In this sense, it is important to differentiate two optimisation strategies: (1) hydrothermal hydrolysation of HBSG to obtain a higher RS yield and (2) FHP in *E. coli*. Although OP5 is a promising condition, HMF is significantly produced with temperatures higher than 132°C.

Therefore, to evaluate the viability of hydrolysated BSGs, depending on free carbohydrates, proteins, and the likely negative effect of HMF for FHP in *E. coli*, the A17 HBSG was used as the HBSG-based medium in 20% and 40% (v/v). As previously mentioned, the HBSG A16 showed the highest RS and protein concentrations; in A17f, the Y_VH2_ was higher (48 mmol/L) than in A15f and A16f. This result may be due to the low HMF concentration detected (<0.001 mg/L) in contrast to 10–16 mg/L found in A15f and A16f, respectively (Table 4). It is important to quantify the production of HMF, among other compounds, to determine the optimal conditions for fermentative processes to avoid producing toxic by-products. Therefore, depending on the application of hydrolysated BSG and the limited conditions of H_2_SO_4_ acid or temperature, any of these conditions could be feasible in terms of RS concentrations and yields, in which HMF does not affect its toxicity to the microbiological processes.

FHP was obtained by Poladyan et al. (2018) and Mirzoyan et al. (2020) at concentrations of 1.4–1.5 mmol/L in the *hyaBhybC* double mutant with 4 or 10% HBSG obtaining an external pH 5.2. In this work, the pH values obtained in A15f–A17f were higher than 5.8, so this parameter is important to take into account in future work with *E. coli.* At lower pH values, the H_2_ generation is significantly diminished as the Hyd-1 and 2 are key enzymes in oxidizing H_2_.

It is worth noting that acetate has been produced in the three HBSG samples tested, although in A17, succinate has not been produced (Table 4). The VFA makes it possible to use the fermentative HBSG for a subsequent fermentation process, for instance, by the photosynthetic bacteria *R. capsulatus* that could assimilate acetate in a photofermentation process. However, it could be very interesting to improve the dark-fermentation process to further enhance carbohydrate assimilation, of which approximately 50% remains in the supernatant. For instance, *E. coli* could be metabolically engineered to obtain a superior strain that could enhance this fermentation step. Future work evaluating the real costs of energy and acid solvents, using a life-cycle assessment (LCA), should be carried out to evaluate the most efficient pre-treatment BSG and its application for the production of bio-products in a large-scale industrial application. Finally, a promising strategy for biotechnological H_2_ production is the design of an integrative system of BSG pre-treatment together with its valorisation for FHP and fermentative end-products in a two-system integration of dark fermentation and photofermentation. It is important to take a sustainable approach to production engineering by implementing system solutions based on the fundamentals of a circular economy.

## Data Availability

The original contributions presented in the study are included in the article/Supplementary Material; further inquiries can be directed to the corresponding author.
