# Supplementary material for: Revalorisation of brewer’s spent grain for biotechnological production of hydrogen with Escherichia coli
Source: Front Bioeng Biotechnol. 2024 Nov 25;12:1473704. doi: 10.3389/fbioe.2024.1473704 (PMC11625585; doi:10.3389/fbioe.2024.1473704)
Supplement: Supplementary file 1 [file DataSheet1.docx]

Supplementary Material

# Supplementary Data

Supplementary Material should be uploaded separately on submission. Please include any supplementary data, figures and/or tables.

Supplementary material is not typeset so please ensure that all information is clearly presented, the appropriate caption is included in the file and not in the manuscript, and that the style conforms to the rest of the article.

# Supplementary Figures and Tables

For more information on Supplementary Material and for details on the different file types accepted, please see [here](https://www.frontiersin.org/guidelines/author-guidelines#supplementary-material).

## Supplementary Figures

**Figure S1.** Cell dry weight per L (CDW/L) of fermentation experiments with 17 different HBSGs at 20% (A) and 40% v/v (B), at 0 h and at 70 h since inoculation.

**Figure S2.** Total protein concentration and free amino acids determination by Lowry and ninhydrin method respectively before and after fermentation of A15, A16 and A17 40% HBSG based medium

## Supplementary Tables

**Table S1.** Determination of total dry weight (DW) of BSG (25 g, 105 º C, 72 h) and moisture of BSG after dried for experiments (DE) (25 g, 50 ºC, 48 h)

|  | Wet Weight (g) | | DW (g) | | DW (%) |
| --- | --- | --- | --- | --- | --- |
|  | **0 h** | **24 h** | **48 h** | **72 h** | **(w/w)** |
| DW1 (105 ºC) | 25 | 17.71 | 17.76 | 17.78 | 71.12% |
| DW2 (105 ºC) | 25 | 17.75 | 17.78 | 17.78 | 71.12% |
| DW3 (105 ºC) | 25 | 17.84 | 17.9 | 17.91 | 71.64% |
|  |  |  |  |  | **71.29%** |
| DE1 (50 ºC) | 25 | 17.01 | 17.07 | - | 68.28% |
| DE2 (50 ºC) | 25 | 16.98 | 17.04 | - | 68.16% |
| DE3 (50ºC) | 25 | 16.95 | 17.02 | - | 68.08% |
|  |  |  |  |  | **68.17%** |

**Table S2.** Particle size fraction of BSG after dried (n=4)

| **P= Size particle (µm)** | **Fraction of BSG (g)** | **Fraction (%)** |
| --- | --- | --- |
| 1700>P>710 | 63.22 | 25% |
| 710>P>355 | 108.76 | 44% |
| P<355 | 77.95 | 31% |

**Table S3.** Pre-treatment with acid and alkali solutions of BSG and contents of reducing sugar (RS, g/L) and proteins (g/L) before and after the pre-treatment. S1, S2 and S3: 0.1, 0.2 and 0.3 M H_2_SO_4_ respectively; C1, C2 and C3: 0.1, 0.2 and 0.3 M HCl; N1, N2 and N3: 0.1, 0.2 and 0.3 M NaOH; K1, K2 and K3: 0.1, 0.2 and 0.3 M KOH and A: water. This table is plotted in Figure 1.

| **Assay** | **RS before (g/L)** | **RS after (g/L)** | **Prot before (g/L)** | **Prot after (g/L)** |
| --- | --- | --- | --- | --- |
| **S1** | 6.75 | 43.73 | 0.43 | 12.71 |
| **S2** | 7.94 | 48.75 | 0.49 | 12.59 |
| **S3** | 8.66 | 48.27 | 0.54 | 13.17 |
| **C1** | 6.75 | 44.08 | 0.86 | 16.66 |
| **C2** | 8.66 | 51.03 | 0.81 | 16.05 |
| **C3** | 7.47 | 51.38 | 0.54 | 14.36 |
| **N1** | 1.12 | 9.86 | 0.02 | 15.15 |
| **N2** | 2.92 | 2.68 | 2.20 | 21.01 |
| **N3** | 1.72 | 1.24 | 2.43 | 26.53 |
| **K1** | 9.86 | 10.10 | 4.17 | 14.57 |
| **K2** | 6.63 | 4.47 | 4.55 | 19.16 |
| **K3** | 7.70 | 1.84 | 5.25 | 25.05 |
| **A** | 7.94 | 11.18 | 0.66 | 2.49 |

**Table S4.** Calculation of acid costs

|  | **MW** | **Density** | **Purity** | **mL acid** | **Price** | **Price** | **Price** |
| --- | --- | --- | --- | --- | --- | --- | --- |
|  | **g/mol** | **g/mL** | **g acid/100 g sol** | **Sol 5M** | **(€/L)** | **(€/mL acid 5M)** | **(€/25 L bottle)** |
| **H_2_SO_4_*** | 98 | 1.84 | 0.96 | 277.40 | 8.21 | 0.002278 | 205.34 |
| **HCl**** | 36.46 | 1.18 | 0.37 | 417.54 | 18.89 | 0.007889 | 472.37 |

*https://customers.scharlab.com/articulo/40/255/7003?xarticulo_id=AC2065025P

**https://customers.scharlab.com/articulo/40/255/9280?xarticulo_id=AC0737025P

**Table S5.** Full factorial analysis 2k using 4 factors as described in table 2. A: autoclave. BSG: brewery spent grain. Carb.: the sum of carbohydrates analysed by HPLC (D-glucose, maltose, D-xylose and D-arabinose). RS: reducing sugar. Prot: total protein. Protein yield: refers to total protein analysed per g BSG hydrolysated. RS yield: g of RS per g BSG added. A17, A18 and A19 conditions are replicates and are considered as central points of DoE.

| **Assay** | **[H_2_SO_4_]** **(M)** | **BSG (%w/v)** | **Time (min)** | **Tª (ºC)** | **RS g/L** | **Carb. (g/L)** | **RS yield (g/g)** | **g RS/g BSG·mL acid** | **Prot (g/L)** | **Protein yield (g/g)** | **HMF (mg/L)** |
| --- | --- | --- | --- | --- | --- | --- | --- | --- | --- | --- | --- |
| **A1** | 0.05 | 5 | 10 | 102 | 5.76 | 7.12 | 0.12 | 0.04 | 0.54 | 0.01 | <0.001 |
| **A2** | 0.15 | 5 | 10 | 102 | 10.70 | 10.49 | 0.21 | 0.03 | 0.65 | 0.01 | <0.001 |
| **A3** | 0.05 | 15 | 10 | 102 | 24.18 | 21.91 | 0.16 | 0.06 | 1.44 | 0.01 | <0.001 |
| **A4** | 0.15 | 15 | 10 | 102 | 36.94 | 23.89 | 0.25 | 0.03 | 2.34 | 0.02 | <0.001 |
| **A5** | 0.05 | 5 | 30 | 102 | 7.20 | 8.93 | 0.14 | 0.05 | 3.94 | 0.08 | <0.001 |
| **A6** | 0.15 | 5 | 30 | 102 | 14.19 | 14.60 | 0.28 | 0.03 | 7.75 | 0.16 | <0.001 |
| **A7** | 0.05 | 15 | 30 | 102 | 25.38 | 24.07 | 0.17 | 0.06 | 9.66 | 0.06 | <0.001 |
| **A8** | 0.15 | 15 | 30 | 102 | 41.88 | 36.25 | 0.28 | 0.03 | 17.36 | 0.12 | <0.001 |
| **A9** | 0.05 | 5 | 10 | 132 | 19.12 | 18.93 | 0.38 | 0.14 | 8.26 | 0.17 | <0.001 |
| **A10** | 0.15 | 5 | 10 | 132 | 25.62 | 22.46 | 0.51 | 0.06 | 8.73 | 0.17 | 1.27 |
| **A11** | 0.05 | 15 | 10 | 132 | 43.32 | 34.53 | 0.29 | 0.10 | 20.27 | 0.14 | 1.93 |
| **A12** | 0.15 | 15 | 10 | 132 | 74.26 | 57.78 | 0.50 | 0.06 | 22.72 | 0.15 | <0.001 |
| **A13** | 0.05 | 5 | 30 | 132 | 20.33 | 19.66 | 0.41 | 0.15 | 8.33 | 0.17 | 0.43 |
| **A14** | 0.15 | 5 | 30 | 132 | 26.23 | 20.37 | 0.52 | 0.06 | 9.81 | 0.20 | 1.60 |
| **A15** | 0.05 | 15 | 30 | 132 | 51.63 | 29.20 | 0.34 | 0.12 | 21.03 | 0.14 | 10.89 |
| **A16** | 0.15 | 15 | 30 | 132 | 80.88 | 56.23 | 0.54 | 0.06 | 24.45 | 0.16 | 15.94 |
| **A17** | 0.1 | 10 | 20 | 117 | 35.98 | 31.55 | 0.36 | 0.06 | 15.35 | 0.15 | <0.001 |
| **A18** | 0.1 | 10 | 20 | 117 | 36.34 | 23.14 | 0.36 | 0.07 | 14.70 | 0.15 | <0.001 |
| **A19** | 0.1 | 10 | 20 | 117 | 36.22 | 22.78 | 0.36 | 0.07 | 15.45 | 0.15 | <0.001 |

**Table S6.** Fiber analysis of raw BSG and the solid generated from hydrolyzation of BSG with water and thermal hydrolysation with sulphuric acid, that represent the relative concentration of each component (%) in each fraction following the FIBER method described in material and method section 2.6.

|  | **Before treatment** | **After treatment** | | |
| --- | --- | --- | --- | --- |
| **Components of BSG** | **Raw BSG (%)** | **Solids HBSG + water (%)** | **Solids**  **HBSG A15 (%)** | **Solids**  **HBSG A16 (%)** |
| Lipids | 2.91 | 3.07 | 11.15 | 14.59 |
| Proteins | 69.79 | 23.32 | **21.00** | **20.81** |
| Hemycellulose | 19.79 | 40.55 | **10.79** | **8.20** |
| Cellulose and soluble lignin | 6.65 | 21.68 | 39.00 | 38.74 |
| Insoluble lignin | 0.86 | 11.37 | 18.07 | 17.66 |
| Insoluble salts | 2.99 | 1.89 | 2.87 | 2.47 |
| **TOTAL** | **100** | **100** | **100** | **100** |
| Solid recovery | 100 | 59.8 | 42.2 | 41.9 |

**Table S7.** Optimization of ascendent rate by increment of temperature concomitantly with decrement of sulfuric acid concentration. Assays were performed in a stainless-steel reactor (R). R2 was performed with the same conditions as done in autoclave (A15). Concentration of protein. RS: reducing sugar, Prot: total protein. Carb: sum of carbohydrates (MGAX) in g/L analysed by HPLC.

| **Assays** | **[H_2_SO_4_] M** | **Tª (ºC)** | **RS g/L** | **Prot (g/L)** | **Carb. (g/L)** | **g RS/g BSG** | **g RS/mL acid** | **HMF (mg/L)** |
| --- | --- | --- | --- | --- | --- | --- | --- | --- |
| **R1** | 0.06 | 117 | 36.29 | 15.00 | 18.38 | 0.24 | 10.90 | 7.8 |
| **R2 (=A15)** | 0.05 | 132 | 47.13 | 22.70 | 26.01 | 0.31 | 16.99 | 4.7 |
| **R3** | **0.04** | **147** | **48.99** | **18.39** | **37.00** | **0.33** | **22.08** | 29.9 |
| **R4** | 0.03 | 162 | 41.32 | 15.65 | 30.28 | 0.28 | 24.83 | 49.8 |
| **R5** | 0.02 | 177 | 31.52 | 17.45 | 23.47 | 0.21 | 28.40 | 211.1 |
| **R6** | 0.01 | 192 | 22.71 | 17.48 | 13.17 | 0.15 | 40.93 | 281.8 |
| **R7** | 0 | 207 | 4.66 | 0.00 | 2.26 | 0.03 | 0 | 171.3 |

**Table S8.** Optimization by Central Composite Design (CCD) for k=2 using the parameters of sulfuric acid concentration and temperature. Central points (0,0) for H_2_SO_4_ is: 0.04 M and temperature: 147ºC. The response variables are: reducing sugar (RS) and proteins (Prot) both in (g/L). In asterisk are indicated the conditions that are performed in autoclave. The rest of the conditions were performed in stainless reactor.

| **Assay** | **A** | **D** | **[H_2_SO_4_] M** | **Tª (ºC)** | **RS (g/L)** | **Prot (g/L)** | **HMF (mg/L)** |
| --- | --- | --- | --- | --- | --- | --- | --- |
| **OP1 (=R2)*** | -1 | -1 | 0.050 | 132.0 | 46.26 | 17.6 | 4.7 |
| **OP2*** | 1 | -1 | 0.030 | 132.0 | 27.76 | 14.5 | 2.3 |
| **OP3** | -1 | 1 | 0.050 | 162.0 | 59.39 | 18.6 | 172.6 |
| **OP4 (=R4)** | 1 | 1 | 0.030 | 162.0 | 41.32 | 18.4 | 34.1 |
| **OP5** | -1.41 | 0.00 | 0.054 | 147.0 | 55.78 | 18.6 | 22.3 |
| **OP6** | 1.41 | 0.00 | 0.026 | 147.0 | 38.75 | 18.6 | 144.0 |
| **OP7*** | 0.00 | -1.41 | 0.040 | 125.8 | 31.22 | 15.8 | 24.0 |
| **OP8** | 0.00 | 1.41 | 0.040 | 168.2 | 47.19 | 16.5 | 21.7 |
| **OP9** | 0 | 0 | 0.040 | 147.0 | 48.99 | 16.0 | 2.3 |
| **OP10** | 0 | 0 | 0.040 | 147.0 | 48.00 | 24.7 | 172.6 |
| **OP11 (=R3)** | 0 | 0 | 0.040 | 147.0 | 48.99 | 22.7 | 34.1 |

**Table S9.** Carbohydrates detected by HPLC at initial and after fermentation of 17 HBSGs. M: average. SD: standard deviation.

|  | **D-glucose** | | | **D-xylose** | | | **Maltose** | | | **D-arabinose** | | |
| --- | --- | --- | --- | --- | --- | --- | --- | --- | --- | --- | --- | --- |
| **HBSG**  **Conditions 40 %** | **M**  **0 h** | **M**  **70 h** | **SD** | **M**  **0 h** | **M**  **70 h** | **SD** | **M**  **0 h** | **M**  **70 h** | **SD** | **M**  **0 h** | **M**  **70 h** | **SD** |
| A1 | 0.46 | 0.14 | 0.02 | 0.21 | 0.08 | 0.00 | 3.13 | 2.08 | 0.27 | 0.72 | 0.52 | 0.00 |
| A2 | 1.03 | 1.96 | 2.45 | 0.55 | 0.11 | 0.00 | 3.23 | 2.45 | 0.36 | 0.81 | 0.54 | 0.19 |
| A3 | 0.59 | 0.24 | 0.06 | 0.30 | 0.19 | 0.01 | 6.97 | 3.97 | 0.58 | 1.13 | 0.95 | 0.05 |
| A4 | 2.30 | 0.39 | 0.07 | 1.16 | 0.28 | 0.01 | 6.75 | 4.80 | 0.54 | 2.70 | 2.32 | 0.05 |
| A5 | 0.63 | 0.16 | 0.02 | 0.31 | 0.10 | 0.00 | 3.03 | 2.77 | 0.65 | 0.76 | 0.66 | 0.00 |
| A6 | 1.68 | 0.20 | 0.04 | 1.24 | 0.12 | 0.00 | 3.05 | 2.73 | 0.61 | 0.97 | 0.78 | 0.02 |
| A7 | 0.54 | 0.23 | 0.03 | 0.40 | 0.18 | 0.01 | 3.80 | 3.66 | 0.38 | 1.28 | 1.03 | 0.20 |
| A8 | 2.85 | 0.33 | 0.04 | 2.03 | 0.22 | 0.01 | 5.95 | 4.20 | 0.47 | 2.25 | 1.82 | 0.03 |
| A9 | 2.63 | 0.19 | 0.02 | 2.09 | 0.13 | 0.01 | 2.78 | 2.74 | 0.60 | 1.13 | 0.90 | 0.02 |
| A10 | 4.46 | 0.22 | 0.04 | 2.97 | 0.37 | 0.10 | 1.63 | 2.06 | 0.57 | 1.23 | 1.00 | 0.01 |
| A11 | 2.85 | 0.46 | 0.06 | 2.19 | 0.37 | 0.01 | 8.25 | 5.72 | 0.50 | 3.22 | 2.83 | 0.05 |
| A12 | 10.38 | 1.59 | 0.03 | 7.77 | 6.74 | 0.19 | 3.55 | 3.87 | 0.53 | 3.49 | 3.06 | 0.11 |
| A13 | 3.04 | 0.13 | 0.01 | 2.20 | 0.10 | 0.00 | 2.01 | 2.33 | 0.64 | 0.98 | 0.69 | 0.00 |
| A14 | 4.72 | 0.23 | 0.05 | 3.50 | 0.15 | 0.01 | 1.44 | 1.91 | 0.62 | 1.37 | 0.97 | 0.03 |
| A15 | 7.82 | 0.21 | 0.14 | 3.04 | 2.12 | 0.46 | 9.46 | 6.80 | 4.77 | 2.63 | 2.26 | 0.71 |
| A16 | 9.39 | 0.33 | 0.00 | 3.69 | 1.05 | 1.06 | 2.16 | 5.73 | 3.07 | 1.41 | 2.32 | 0.58 |
| A17 | 3.41 | 0.28 | 0.01 | 3.03 | 0.25 | 0.01 | 4.32 | 4.57 | 0.03 | 1.99 | 2.27 | 0.01 |

**Table S10.** Volatile Fatty acids (acetate and succinate) and ethanol detected at initial time and after fermentation of 17 different HBSGs.

|  | **Acetato** | | | **Ethanol** | | | **Succinate** | | |
| --- | --- | --- | --- | --- | --- | --- | --- | --- | --- |
| **HBSG Conditions 40 %** | **M**  **0 h** | **M**  **70 h** | **SD** | **M**  **0 h** | **M**  **70 h** | **SD** | **M**  **0 h** | **M**  **70 h** | **SD** |
| A1 | 0.19 | 1.49 | 0.02 | 2.83 | 4.23 | 2.39 | 0.04 | 0.09 | 0.00 |
| A2 | 0.16 | 1.74 | 0.25 | 3.96 | 2.76 | 0.23 | 0.02 | 0.16 | 0.00 |
| A3 | 0.07 | 2.64 | 0.22 | 3.67 | 4.71 | 0.44 | 0.04 | 0.58 | 0.00 |
| A4 | 0.50 | 4.05 | 0.18 | 4.32 | 5.17 | 0.14 | 0.09 | 0.83 | 0.01 |
| A5 | 0.16 | 1.66 | 0.06 | 1.31 | 1.37 | 0.12 | 0.04 | 0.11 | 0.00 |
| A6 | 0.19 | 2.36 | 0.10 | 6.43 | 6.45 | 0.31 | 0.05 | 0.49 | 0.02 |
| A7 | 0.11 | 2.76 | 0.16 | 2.08 | 6.13 | 0.06 | 0.00 | 0.54 | 0.03 |
| A8 | 0.44 | 3.64 | 0.07 | 4.82 | 5.42 | 0.10 | 0.10 | 0.97 | 0.07 |
| A9 | 0.20 | 2.83 | 0.16 | 3.13 | 4.01 | 0.18 | 0.07 | 0.76 | 0.03 |
| A10 | 0.24 | 3.20 | 0.12 | 2.63 | 3.94 | 0.45 | 0.10 | 1.71 | 0.09 |
| A11 | 0.53 | 4.30 | 0.13 | 3.55 | 4.33 | 0.13 | 0.14 | 1.44 | 0.08 |
| A12 | 0.67 | 3.19 | 0.24 | 2.79 | 3.41 | 0.14 | 0.23 | 4.71 | 0.01 |
| A13 | 0.25 | 2.56 | 0.10 | 3.16 | 3.58 | 0.14 | 0.08 | 0.77 | 0.04 |
| A14 | 0.31 | 3.36 | 0.16 | 5.14 | 6.12 | 0.35 | 0.11 | 1.61 | 0.05 |
| A15 | 0.84 | 2.84 | 0.55 | 6.77 | 7.19 | 3.01 | 0.34 | 2.97 | 0.33 |
| A16 | 0.33 | 2.90 | 1.05 | 2.92 | 6.15 | 2.28 | 0.23 | 3.02 | 0.10 |
| A17 | 0.44 | 3.54 | 0.06 | 3.25 | 5.99 | 0.34 | 0.10 | 0.21 | 0.02 |
